# EIGER detector: application in macromolecular crystallography

**DOI:** 10.1107/S2059798316012304

**Published:** 2016-08-31

**Authors:** Arnau Casanas, Rangana Warshamanage, Aaron D. Finke, Ezequiel Panepucci, Vincent Olieric, Anne Nöll, Robert Tampé, Stefan Brandstetter, Andreas Förster, Marcus Mueller, Clemens Schulze-Briese, Oliver Bunk, Meitian Wang

**Affiliations:** a Swiss Light Source, Paul Scherrer Institute, 5232 Villigen, Switzerland; b Institute of Biochemistry, Biocenter, Goethe University Frankfurt, Max-von-Laue-Strasse 9, 60438 Frankfurt am Main, Germany; c DECTRIS Ltd, Taefernweg 1, 5405 Baden-Dättwil, Switzerland

**Keywords:** X-ray detectors, EIGER detector, macromolecular crystallography, data-collection strategy

## Abstract

The development of single-photon-counting detectors, such as the PILATUS, has been a major recent breakthrough in macromolecular crystallography, enabling noise-free detection and novel data-acquisition modes. The new EIGER detector features a pixel size of 75 × 75 µm, frame rates of up to 3000 Hz and a dead time as low as 3.8 µs. An EIGER 1M and EIGER 16M were tested on Swiss Light Source beamlines X10SA and X06SA for their application in macromolecular crystallography. The combination of fast frame rates and a very short dead time allows high-quality data acquisition in a shorter time. The ultrafine φ-slicing data-collection method is introduced and validated and its application in finding the optimal rotation angle, a suitable rotation speed and a sufficient X-ray dose are presented. An improvement of the data quality up to slicing at one tenth of the mosaicity has been observed, which is much finer than expected based on previous findings. The influence of key data-collection parameters on data quality is discussed.

## Abstract 2

The application of the EIGER hybrid photon-counting pixel detector in macromolecular crystallography is presented. Data-collection strategies exploiting the unique features of EIGER are discussed.

## 1. Introduction   

The huge improvements in the past decade in synchrotron sources, instrumentation at macromolecular crystallography (MX) beamlines, X-ray detectors, and data-processing and structure-determination software (Gruner & Lattman, 2015 ▸; Gruner *et al.*, 2012 ▸; Smith *et al.*, 2012 ▸; Minor *et al.*, 2006 ▸; Kabsch, 2010*a* ▸,*b* ▸; Sheldrick, 2010 ▸; Adams *et al.*, 2010 ▸) have enabled X-ray structure determination of biological macromolecules at an unprecedented pace (http://biosync.sbkb.org). These developments have also called for a revision of the traditional data-collection practice for single crystals using the rotation method, *i.e.* high-dose exposure with minimum redundancy and a coarse rotation increment (typically 0.5–1°), originally designed for imaging plates and later applied to charge-coupled device (CCD) detectors (Dauter, 1999 ▸; Bourenkov & Popov, 2006 ▸). Hybrid photon-counting (HPC) pixel-array detectors, such as the PILATUS, offer several novel features including single-photon sensitivity, a sharp point-spread function of one pixel, millisecond and noise-free readout, and a high dynamic range of 20 bits (Hülsen *et al.*, 2006 ▸). HPC pixel-array detectors enable shutterless data collection in the so-called ‘fine φ-slicing’ mode, improving the data quality and reducing the data-acquisition time (Mueller *et al.*, 2012 ▸). We have demonstrated that these fast and accurate detectors, in combination with novel diffraction goniometry and low-dose high-redundancy data-acquisition schemes, have considerably widened the range of applications for experimental phasing, particularly native SAD, which can now be considered to be a routine method (Weinert *et al.*, 2015 ▸; Liu & Hendrickson, 2015 ▸). New experimental techniques, such as micro-crystallography (Cusack *et al.*, 1998 ▸; Smith *et al.*, 2012 ▸), serial crystallography (Gati *et al.*, 2014 ▸) and room-temperature crystallography (Owen *et al.*, 2014 ▸), continuously present new challenges that require new protocols for obtaining the most accurate and complete data while limiting the effects of radiation damage (Ravelli & Garman, 2006 ▸).

EIGER is a new-generation pixel-array detector with the basic technology being developed at the Paul Scherrer Institute (PSI; Dinapoli *et al.*, 2011 ▸). The PSI EIGER and the DECTRIS EIGER detectors share the same basic technology but have different read-out electronics optimized for different purposes. The DECTRIS version of the EIGER detector has been used in this study and is referred to as EIGER in this paper. The applications of the EIGER detector include macromolecular crystallography (MX), small-angle X-ray scattering (SAXS), coherent diffraction imaging (CDI) and X-ray photon correlation spectroscopy (XPCS) (Yamano *et al.*, 2016 ▸; Johnson *et al.*, 2012 ▸; Dinapoli *et al.*, 2011 ▸; Radicci *et al.*, 2012 ▸). Compared with PILATUS, EIGER features a smaller pixel size (75 × 75 µm), a shorter dead time (as low as 3.8 µs), a higher frame rate (up to 3000 Hz) and a fast 40 Gbit s^−1^ readout. To make full use of the performance of the detector, the network and file system must match. Therefore, file formats that aim at storing one image per file are no longer suitable. To overcome this, EIGER data are stored in the HDF5 format (Hierarchical Data Format; http://hdf5group.org). The HDF5 data model supports data relationships through its grouping and linking mechanisms and stores experimental metadata in the same file structure as the detector data (Mason *et al.*, 2010 ▸).

Here, we present results on the application of EIGER in macromolecular crystallography, obtained with an EIGER 1M and an EIGER 16M on the X10SA and X06SA beamlines of the Swiss Light Source. The new data-acquisition method with ultrafine φ-slicing is demonstrated. The benefits of EIGER’s smaller pixel size, enhanced data-collection speeds and internal summation of ultrafine φ-sliced data are presented. In addition, we demonstrate the accuracy of EIGER data acquired at high angular speed with the successful native SAD phasing of insulin from data collected in only 1 s.

## 2. Materials and methods   

### 2.1. Protein and crystallization   

Insulin was dissolved in 50 m*M* Na_2_HPO_4_, 10 m*M* EDTA pH 10.8 and crystallized in a cryoprotective condition consisting of 25–32% ethylene glycol. Chicken egg-white lysozyme was crystallized in 5% PEG MME 5000, 2 *M* NaCl, 50 m*M* sodium acetate pH 4.5, 25% ethylene glycol. Crystals of TmrAB (*Thermus thermophilus* multidrug-resistance protein A and B) were grown, cryoprotected and snap-cooled in liquid nitrogen (the TmrAB work will be published separately). The TmrAB crystals belong to space group *P*6_5_22 and feature a long *c* axis in the unit cell (*a* = *b* = 93.4, *c* = 1044.0 Å).

### 2.2. EIGER detector characteristics and frame-summation method   

Here, the terms ‘frame’ and ‘image’ have specific meanings. A ‘frame’ refers to a single internal readout in the EIGER detector. An ‘image’ refers to a combination of frames, where the detector and/or computer perform the summation.

#### 2.2.1. Continuous readout, internal frame rate, auto-summation and count rate   

**Figure 1:**
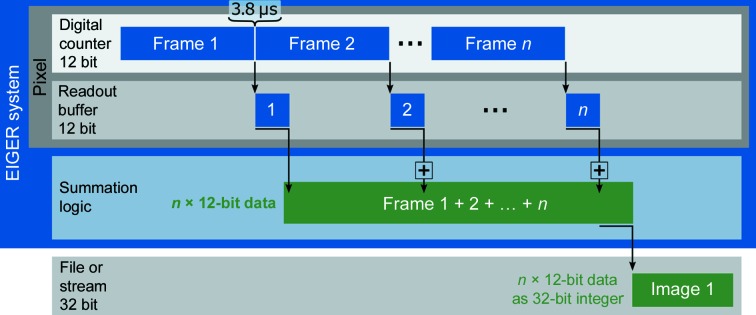
Principle of frame summation. A schematic representation of the readout of an EIGER detector pixel and the auto-summation is depicted. The frames acquired on the digital counter of an EIGER system are transferred to the readout buffer, allowing another frame to be collected after only 3.8 µs. The summation logic that allows the extension of the counter bit-depth is also represented.

One of the hallmark features of the EIGER detector is its continuous readout. Every pixel of an EIGER ASIC (application-specific integrated circuit) features a digital counter for noise-free photon detection and a readout buffer. After the acquisition of a frame, the state of the counter is transferred to the readout buffer. A subsequent frame can start after 3.8 µs (20 µs was used in the EIGER 1M tests presented in this work), while the previous frame is being read out from the readout buffer. All counts are captured in the digital counter of each pixel at high internal frame rates (about 800 Hz for EIGER 4M, 9M and 16M and 3000 Hz for EIGER 1M). A single frame is limited to the 12 bits (4096 counts) of the digital counter, and subsequent summation of frames to images can extend the data depth up to 32 bits or 4.3 billion counts per pixel, depending on the number of summed frames. Even if lower image rates are requested, internally frames are still acquired at high rates at the pixel level, which effectively avoids overflow of the digital counter and extends the bit depth of the data by the number of summed frames (Fig. 1 ▸). This process is called auto-summation and is performed in a user-transparent manner, and is similar to the concept used in X-ray television detectors (Arndt & Gilmore, 1979 ▸). The resulting duty cycle of EIGER is greater than 99% regardless of the externally requested image rate. Here, the duty cycle is defined as the proportion of time during which the detector is counting photons to the exposure time of the image. The maximum external image rate is limited by the data-transfer bandwidth between the detector and the detector-control unit (DCU), which is 3000, 750, 238 and 133 Hz for EIGER 1M, 4M, 9M and 16M, respectively. The auto-summation mode was disabled for one test where 0.05 s exposures were collected as single internal frames (§[Sec sec3.2.1]3.2.1).

**Figure 2:**
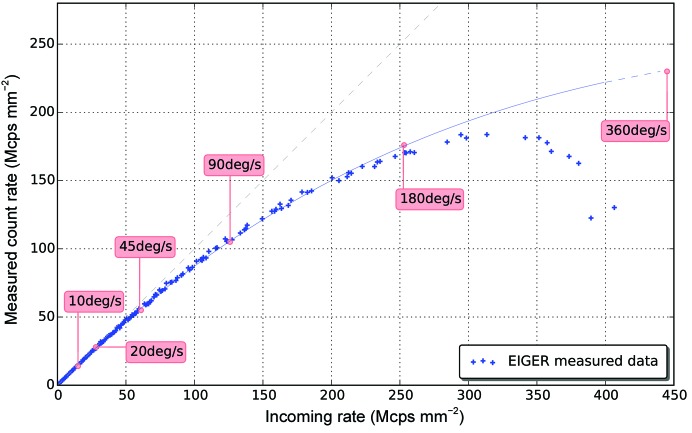
EIGER count-rate performance. The measured count rate is plotted against the incoming rate for 12.4 keV X-rays. The solid line is a fit to the measured data with the equation *I*_obs_ = *I*_0_exp(−*I*_0_ × τ), where *I*_obs_ is the detected count rate, *I*_0_ is the true incident count rate and τ is the energy-dependent dead time of the counter. On this plot, an estimation of the count rate of a reflection with 10 photons for each of the different speed data sets (see §[Sec sec3.3]3.3 and Fig. 9 ▸) is marked.

The EIGER count-rate performance is plotted in Fig. 2 ▸. At count rates of above 50 Mcps mm^−2^ (where Mcps is a million counts per second), EIGER’s counter starts to deviate from a linear response. This deviation is owing to the ‘paralyzable counter’ effect; that is, when a pixel is not sensitive to a subsequent arriving photon because the shaping time between the photons is too short (also called the pile-up effect). A count-rate correction is applied by default and the detector delivers the values for the ‘true counts’. The corrections are based on tabulated correction factors derived from a fit to the measured count-rate curve (solid line in Fig. 2 ▸), and at count rates up to 200 Mcps mm^−2^ (1.1 Mcps per pixel) the counter follows the fit function well. Indeed, the internal frame rates of 800–3000 Hz with the 12-bit digital counter ensures that the counter will not overflow before the count-rate limit of up to 2 × 10^6^ photons s^−1^ per pixel (∼350 Mcps mm^−2^) is reached. Owing to the smaller pixel size, the count rate per area in EIGER is comparable with that of PILATUS3, the count-rate limit of which is extended with a retriggering method, which effectively overcomes counter paralyzation by detecting photon pile-up and re-enabling the counting circuit.

#### 2.2.2. External frame summation   

In addition to the auto-summation in EIGER, in-house-developed Python scripts were used to perform both summation and skipping of frames to validate the auto-summation concept as well as assessing the influence of dead time on data quality. The resulting image was obtained by adding the individual pixel values of a defined number of subsequent frames, thus increasing the total rotation angle per summed image. For example, in the case of lys_2 (Table 1 ▸), every 40 frames, each with a rotation angle of 0.00125°, were summed to generate the data set lys_2_SUM40 consisting of images with a rotation angle of 0.05°. By changing the number of summed frames, data sets lys_2_SUM5, lys_2_SUM10, lys_2_SUM20, lys_2_SUM80 and lys_2_SUM160 were obtained to simulate data sets with rotation angles of 0.00625, 0.0125, 0.025, 0.1 and 0.2°, respectively. The ins_1_SUM5 to ins_1_SUM160 data sets were generated in the same way. Frame summation was also combined with the systematic skipping of frames. By adjusting the number of skipped frames, a missing angular coverage of reciprocal space can be simulated. For example, in the case of lys_2_SUM40, the data set lys_2_20SUM1SKIP1 was obtained with half of the data by first skipping every other frame and then summing blocks of 20 remaining frames into one image; the data set lys_2_SUM20SKIP20 summed 20 frames and skipped the next 20. All three data sets have covered the same total rotation range, but both lys_2_SUM20SKIP20 and lys_2_20SUM1SKIP1 lack half of the information. In a similar way, the ins_1 data set was used to generate ins_1_SUM40, ins_1_SUM20SKIP20 and ins_1_20SUM1SKIP1.

### 2.3. Data collection   

EIGER 1M data were collected with a dead time of 20 µs and a frame rate of up to 800 Hz on beamline X10SA at the Swiss Light Source (SLS). EIGER 4M(16M) data were obtained by reading out one quadrant (the region of interest) of the EIGER 16M with 3.8 µs dead time on SLS beamline X06SA. The threshold energy of both detectors was set to half of the X-ray energy in all experiments to achieve a point-spread function of a single pixel (Broennimann *et al.*, 2006 ▸). All data were collected at 100 K using a cold nitrogen stream. Except for the native SAD phasing experiment, data from lysozyme and insulin crystals were collected with an X-ray beam size of 50 × 30 µm at 12.0 keV (1.0332 Å).

A summary of the most important data-collection and processing statistics for all data sets can be found in Tables 1 ▸ and 2 ▸. The lysozyme data were collected from a crystal of approximately 200 × 100 × 50 µm with a detector distance of 50 mm. At the same crystal position and using the same starting angle, 180° of data were collected in two ways: one fine φ-sliced data set (named lys_1, consisting of 0.05°/0.05 s per frame) and one ultrafine φ-sliced data set (lys_2, 0.00125°/0.00125 s per frame). For the lys_1 data set, the internal summation of the EIGER detector was inactivated in order to obtain single frames of 0.05°. This reduces the available bit depth of the counter. To avoid overloading of the 12-bit counter the beam transmission was set to 0.2%, which corresponds to a flux of 7.2 × 10^9^ photon s^−1^. The accumulated X-ray dose of each data set was estimated at about 0.20 MGy and the mosaicity, as estimated by *XDS*, was 0.23°. Another spot on the same lysozyme crystal was used for data collection at high angular speed (Table 2 ▸). In this case, a series of 180° data sets were collected with an exposure time per frame of 0.00125 s (800 Hz) and with rotations ranging between 0.00125 and 0.9°, *i.e.* corresponding to speeds ranging from 1 to 720° s^−1^ (data sets lys_3 to lys_10). Each data set received the same total dose of 0.1 MGy, which was insufficient to cause significant radiation damage (data not shown).

The insulin data set ins_1 was collected from a crystal of approximately 150 × 100 × 50 µm. 90° of data were collected with a beam attenuated to 0.5% (1.8 × 10^10^ photon s^−1^), with the detector distance set to 60 mm and with a 0.00125°/ 0.00125 s strategy (data set ins_1). The accumulated dose was 0.25 MGy.

For the experiment on TrmAB crystals, the X-ray beam was focused to 50 × 10 µm at the sensor of the EIGER detector to minimize the diffraction spot size for maximum spot separation, and beam-defining slits were used to make a 100 × 100 µm sized beam matching the size of the TrmAB crystals. Diffraction patterns were collected at a detector distance of 300 mm.

The insulin native SAD experiment (data set ins_2 in Table 1 ▸) was performed on a crystal measuring 200 × 200 × 100 µm with a 80 × 30 µm X-ray beam at 8.0 keV (1.5498 Å), which was selected as a compromise between anomalous signal strength and recordable diffraction resolution. At a distance of 130 mm, we obtained a resolution of 2.8 Å at the edges of the EIGER 4M(16M) detector. A data set of 64° was collected at a frame rate of 160 Hz with a rotation range of 0.4° (*i.e.* 0.00625 s exposure time per image). The total exposure time was 1 s. With an 80 × 30 µm sized beam and a flux of 1 × 10^12^ photon s^−1^, the accumulated dose was estimated to be 0.5 MGy.

### 2.4. Data processing and analysis   

All data sets were processed using the *XDS* package (Kabsch, 2010*a* ▸,*b* ▸). The scaling statistics are reported as calculated by the CORRECT step in *XDS*. The definition of mosaicity used in this paper is, as described in *XDS*, the standard deviation of the reflection profiles assuming a Gaussian distribution. The X-ray dose was estimated based on equation (5) in Holton (2009 ▸). For the insulin native SAD structure, experimental phasing was carried out with *SHELXC*/*D*/*E* (Sheldrick, 2010 ▸) *via* the *HKL*2*MAP* interface (Pape & Schneider, 2004 ▸), and iterative model building, density modification and refinement were carried out with *Buccaneer* (Cowtan, 2012 ▸), *Parrot* (Cowtan, 2010 ▸) and *REFMAC*5 (Murshudov *et al.*, 2011 ▸) *via* the *CRANK*2 pipeline (Skubák & Pannu, 2011 ▸, 2013 ▸). The lysozyme and insulin structures were refined with *phenix.refine* (Afonine *et al.*, 2012 ▸).

The coordinates and diffraction data have been deposited in the Protein Data Bank (PDB) as entries 5lin for the lysozyme 1° s^−1^ structure, 5lio for the lysozyme 360° s^−1^ structure and 5lis for the insulin 1 s native SAD structure.

## 3. Results and discussion   

### 3.1. Advantages of small pixel size   

**Figure 3:**
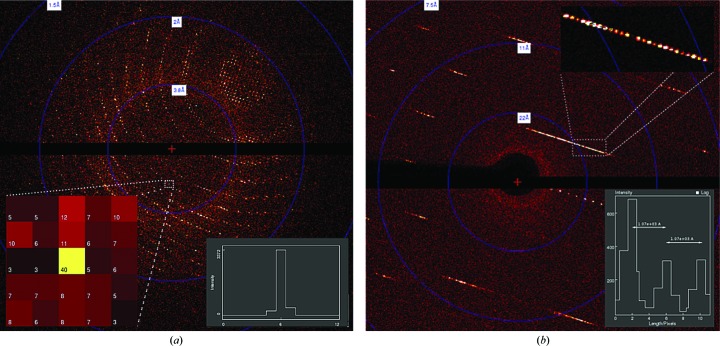
Diffraction images obtained with the EIGER 1M. (*a*) Diffraction pattern of a lysozyme crystal. A close-up view and the cross-section plot where a spot is recorded exclusively on one pixel are shown. (*b*) Diffraction pattern of a TmrAB crystal; the close-up displays the clear separation of spots and the spacing between spots indicates the unit-cell axis of 1070 Å.

**Figure 4:**
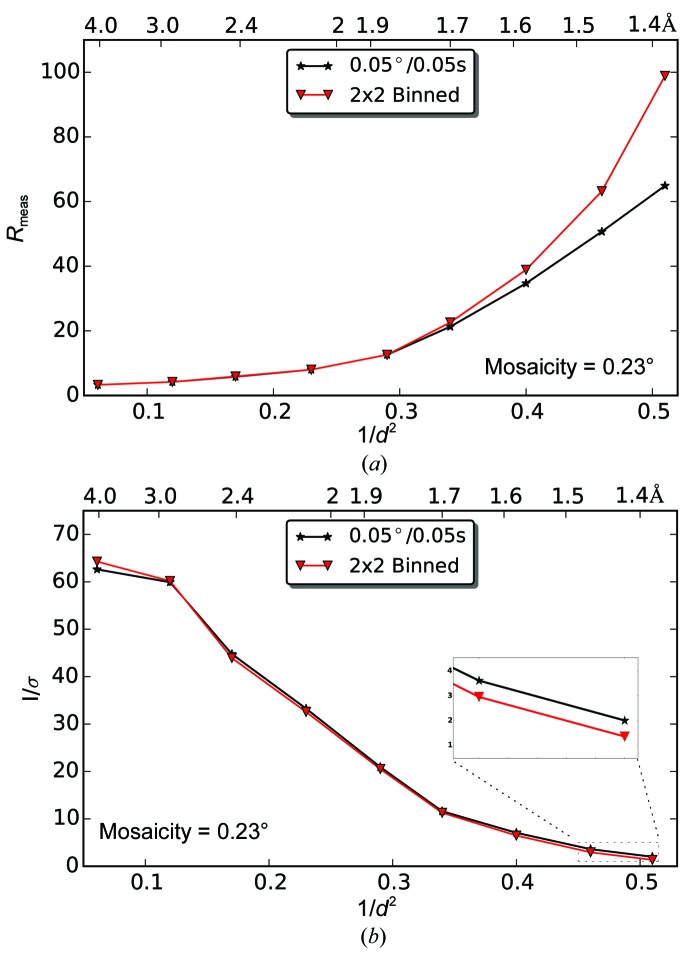
Effect of 2 × 2 binning on EIGER 1M data, corresponding to data collection with detector pixels that are four times larger. Comparison of a lysozyme data set (lys_1) collected with an EIGER 1M with the same data set with all of the diffraction images 2 × 2 binned. Both *R*_meas_ and *I*/*σ*(*I*) are plotted *versus* resolution shells as reported in the CORRECT.LP file of *XDS*. The close-up in (*b*) shows the differences in the two highest resolution shells, where the 2 × 2 binning diminishes the data quality.

A diffraction pattern of a lysozyme crystal collected with the EIGER 1M is shown in Fig. 3 ▸(*a*). Some spots are recorded on a single pixel (75 × 75 µm) as seen in the close-up view and in the cross-section plot. The combination of the small pixel size and the sharp, single-pixel point-spread function enables the measurement of diffraction spots with low background noise, which improves the signal-to-noise ratio of the integrated intensity. This was convincingly demonstrated by comparing the data quality of the EIGER data set lys_1 with 2 × 2 binned images of the same data set to simulate a pixel with four times the area (Fig. 4 ▸ and Supplementary Table S1). The binning has no effect on low- to medium-resolution data because the background noise is dwarfed by the strong signal of the diffraction peak and instrumentation errors. However, in the high-resolution shells where the diffraction signal is weak, the increased background counts under the diffraction peak in the binned image do diminish the data quality [*e.g.* a 32% lower *I*/σ(*I*) in the highest resolution shell]. To extend this comparison to the largest format EIGER and PILATUS detectors, one needs to take into account that the EIGER 16M has 55% of the active area of the PILATUS 6M. To achieve an equivalent diffraction resolution, the EIGER 16M needs to be positioned 0.55^1/2^ = 0.74 times closer than the PILATUS 6M. Therefore, the isotropic background per unit area is 1/0.55 = 1.8 times higher, the isotropic background per pixel is (172/75)^2^/1.8 = 2.9 times lower and the solid angle per pixel is four times smaller [(75/172)^2^/0.55^1/2^] for the EIGER 16M than for the PILATUS 6M. The better sampling of diffraction spots and lower background per pixel could offset the increase in the recorded background scattering.

We then tested the spatial resolution of the EIGER 1M by measuring its capacity to separate closely spaced reflections. For this, we collected data from a TmrAB crystal with a unit-cell axis of *c* = 1044.0 Å using 12 keV (1.0332 Å) X-rays focused at the surface of the EIGER 1M detector positioned 300 mm from the sample. In Fig. 3 ▸(*b*), both in the main image and in the close-up view, the reflections along the reciprocal c* axis are clearly defined and well separated. In the horizontal cross-section of consecutive pixels, the peaks correspond to a unit-cell axis of 1044 Å. We note that at this detector distance but with an EIGER 16M detector, the resolution obtained at the edge of the detector would be 2.2 Å. Therefore, with an EIGER 16M it is possible to effectively resolve a large unit-cell axis while capturing high-resolution diffraction.

### 3.2. Ultrafine φ-slicing data collection   

**Figure 5:**
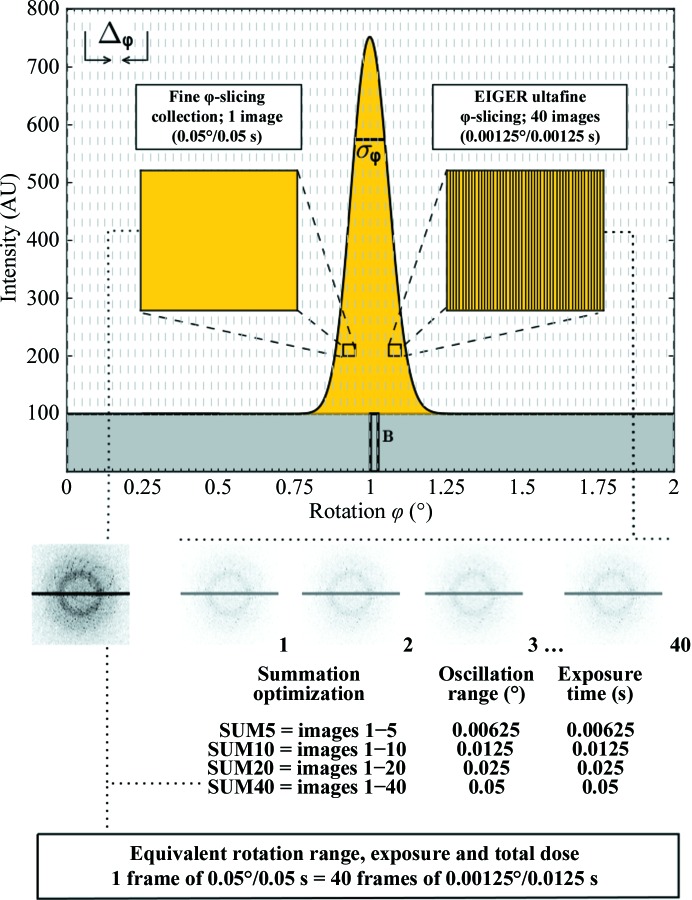
Schematic of both fine φ-slicing and ultrafine φ-slicing data-acquisition methods. A rocking-curve model with the reflection profile along the rotation angle is depicted (Gaussian distribution with σ_φ_ = 0.1°). A comparison is shown of a typical PILATUS 0.05°/0.05 s fine φ-sliced data acquisition on the left with a 800 Hz 0.00125°/0.00125 s EIGER data acquisition on the right. The individual frames in the EIGER data are very weak but when they are summed (40 frames in the example) the same rotation angle, exposure and total dose as for the 0.05°/0.05 s data are obtained.

Ultrafine φ-slicing data collection with EIGER is represented in Fig. 5 ▸, where a rocking-curve model with the background and the reflection profile along the rotation angle (φ) is shown. This represents a Gaussian distribution of the reflection intensity with σ_φ_ = 0.1°. As a comparison, a typical fine φ-slicing data-collection strategy used with the PILATUS detector, where each image corresponds to 0.05°/0.05 s, is depicted on the left (note that the rotation angle is half of the mosaicity). On the right-hand side, an 800 Hz ultrafine φ-slicing data acquisition with EIGER is illustrated. Here, each image corresponds to 0.00125°/0.00125 s. Diffraction spots on any individual frames are very faint, and it is only when they are summed together that a clear diffraction pattern will appear. In this example, adding 40 EIGER frames together will result in a PILATUS-like image with an equivalent rotation range, exposure and total dose. The EIGER data-collection method is only possible owing to the absence of readout noise and the very low dead time. The advantages, which will be discussed further in the following section, are that the ultrafine φ-slicing results in even higher data quality than the current standard fine φ-slicing method, and the high-frame-rate data collection with the EIGER detector enables an optimal choice of the crystal rotation range during an exposure and also in hindsight after collecting data at the highest possible speed.

#### 3.2.1. Validation of EIGER auto-summation   

**Figure 6:**
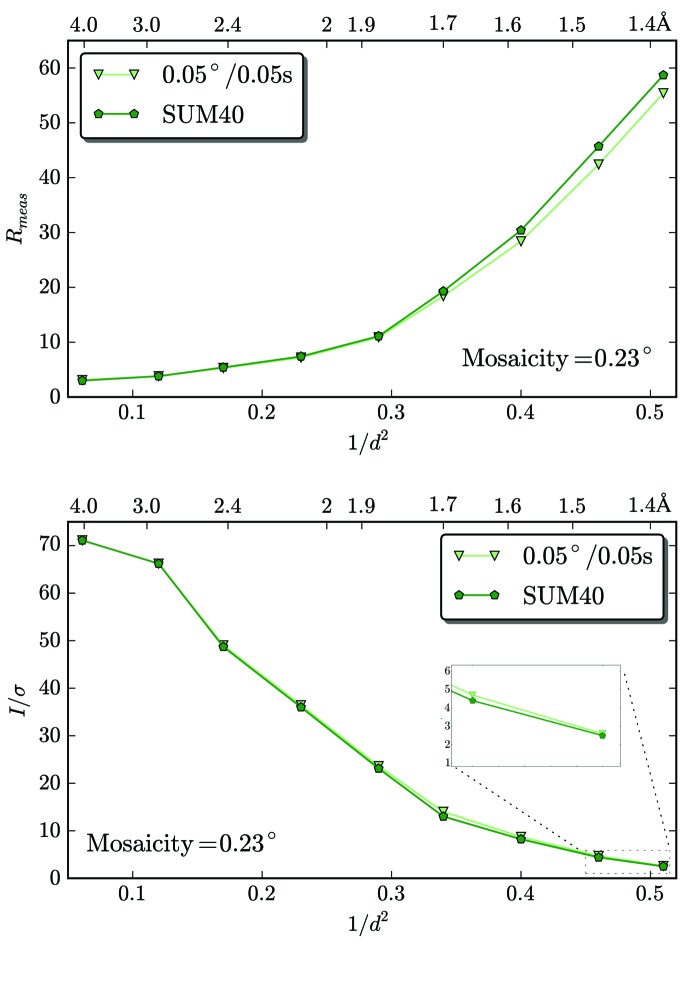
Validation of the EIGER data-acquisition method with internal summation. A comparison of two data sets collected from the same lysozyme crystal is presented. The first data set was collected with 0.05°/0.05 s without internal summation (lys_1). The second data set was collected with 0.00125°/0.00125 s but every 40 images were summed simulating a 0.05° data set (lys_2_SUM40). Both *R*_meas_ and *I*/σ(*I*) indicate that the two data sets have comparable quality.

Two data sets were collected from the same lysozyme crystal with the EIGER detector: one (lys_1) in 0.05°/0.05 s but with EIGER operated in a special low-frame-rate mode without auto-summation (§[Sec sec2.2.1]2.2.1) and the other (lys_2) in 0.00125°/0.00125 s. The X-ray dose rate has been adjusted sufficiently low to avoid overload of the 12-bit counter for the lys_1 data set. Every 40 0.00125°/0.00125 s frames in lys_2 were then summed to simulate 0.05°/0.05 s images. The difference is that the total dead time is 20 µs for the lys_1 data set and 800 µs (40 × 20 µs) for the lys_2 data set. Data-processing statistics are plotted in Fig. 6 ▸ and are listed in Supplementary Table S2. The two data sets are of comparable high quality, with a practically identical CC_1/2_ over the whole resolution range. The *R*_meas_ and *I*/*σ*(*I*) are slightly worse in the higher resolution shells for lys_2. With the even shorter dead time of 3.8 µs in the production model of the EIGER detector, the small difference between data from single-frame images and images summed from many frames is expected to be further minimized.

#### 3.2.2. Optimal rotation angle   

**Figure 7:**
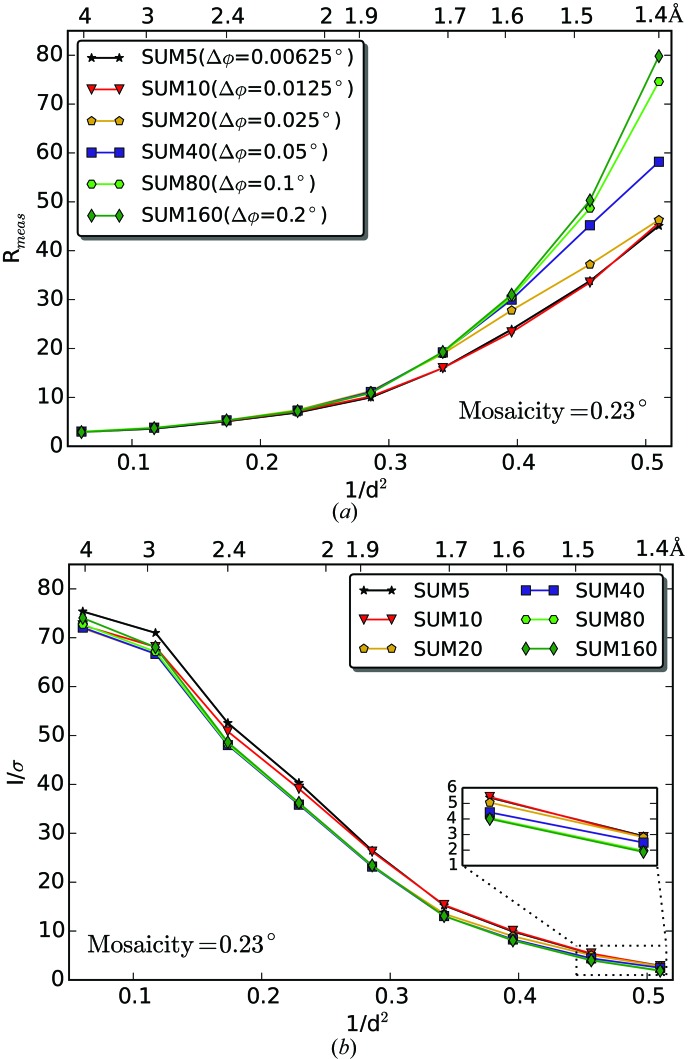
Summation optimization of the EIGER lys_2 data. The lys_2 data set was analyzed by comparing the summation of five (lys_2_SUM5), ten (lys_2_SUM10), 20 (lys_2_SUM20), 40 (lys_2_SUM40), 80 (lys_2_SUM80) and 160 (lys_2_SUM160) frames. In each case, the resulting rotation angle is shown and the effect of the different summations on *R*_meas_ and *I*/σ(*I*) is represented. The lowest *R*_meas_ values are achieved for rotation angles of 1/10 of the mosaicity or below.

The advantages of the fine φ-slicing data-collection strategy have been demonstrated previously both in theory (Pflugrath, 1999 ▸) and with experiments (Mueller *et al.*, 2012 ▸). The general recommendation for PILATUS detectors is to use half of the mosaicity (defined as the r.m.s. of the refined ‘rocking curve’ in *XDS*, which accounts for both the crystal mosaicity and X-ray beam divergence) as the rotation angle per image. For the EIGER, we tested the advantages of ultrafine φ-slicing using lysozyme data sets (lys_2 with a mosaicity of 0.23°) with frames externally summed in various ways. Summations of five (lys_2_SUM5), ten (lys_2_SUM10), 20 (lys_2_SUM20), 40 (lys_2_SUM40), 80 (lys_2_SUM80) and 160 (lys_2_SUM160) frames were carried out to simulate rotation angles of 0.00625, 0.0125, 0.025, 0.05, 0.1 and 0.2°, respectively. The effect of the different summations on the *R*_meas_ and *I*/σ(*I*) at different diffraction resolutions was analyzed (Fig. 7 ▸ and Supplementary Table S3). While lys_2_SUM40 gives very similar statistics to the control data set lys_1 (0.05°/0.05 s), both lys_2_SUM80 and lys_2_SUM160 result in higher *R*_meas_ and lower *I*/σ(*I*) values at high resolution. This is owing to lys_2_SUM80 and lys_2_SUM160 not taking advantage of the fine φ-slicing strategy to reduce the background around the diffraction peak. In contrast to a similar study on PILATUS (Mueller *et al.*, 2012 ▸), we observe that finer slicing below half of the mosaicity can still improve data quality, as seen in data sets lys_2_SUM20, lys_2_SUM10 and lys_2_SUM5, where the rotation angles correspond to about 1/9, 1/18 and 1/37 of the mosaicity, respectively. We note the marginal difference among these three data sets, meaning that there is no further gain upon over sampling the reflection profile along the rotation by more than 1/10 of the mosaicity.

**Figure 8:**
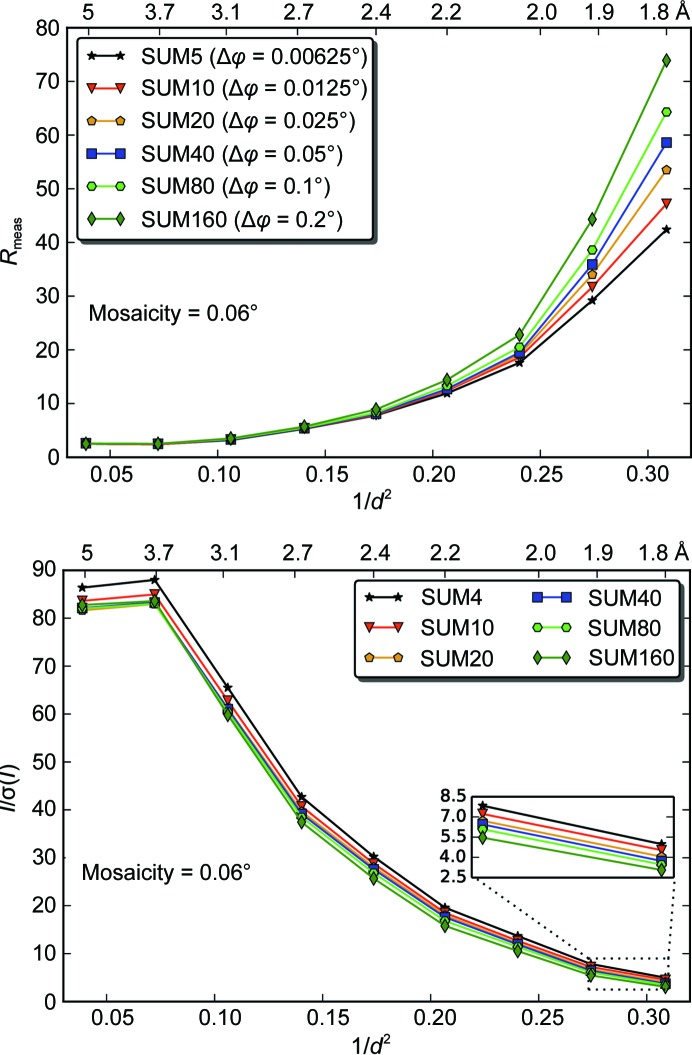
Summation optimization of the EIGER ins_1 data. Ins_1_SUM5 to ins_1_SUM160 were generated. The effect of the different summations on *R*_meas_ and *I*/*σ*(*I*) is represented. The lowest *R*_meas_ value is achieved for an oscillation angle of 1/10 of the mosaicity.

A similar analysis was carried out with the data sets (ins_1_SUM5 to ins_1_SUM160) obtained using a crystal of insulin with a lower mosaicity of 0.06° (Fig. 8 ▸ and Supplementary Table S4). Here, we also observed that the lower the number of frames summed (*i.e.* with finer slicing), the better the *R*_meas_ and *I*/σ(*I*), especially at high resolution. For the ins_1_SUM5 data set, which had the best data quality of the summed data sets, the angular steps of 0.00625° represent about 1/10 of the mosaicity. Note that processing data sets with less than five summed images rendered worse statistics in both test cases (data not shown). We believe this is because the data contained in individual diffraction frames or summed ones are very weak, so the accuracy in locating spot positions and profile fitting during indexing and integration is reduced and the final data quality is compromised. We speculate that data-integration methods adapted to such ultrafine samplings with low counting statistics could improve the quality of the integrated data for low-mosaicity crystals even further (Ayyer *et al.*, 2015 ▸).

Taken together, the results from the summation analysis on both lysozyme and insulin show that an optimal rotation angle for data collection with an EIGER detector is about 1/10 of the mosaicity. The difference between the 1/2 mosaicity rule in the PILATUS study and the current 1/10 mosaicity for EIGER could be related to pixel size and to software advances in three-dimensional peak integration. The larger pixel size of PILATUS results in coarser sampling of the detector space, which will result in wider spot profiles. Therefore, the advantage of the finer angular (φ) sampling, which narrows the width of the spot in the detector space, will not be as pronounced for PILATUS as for EIGER. An additional effect could come from data-processing software that has been optimized for data sets with both spatially and angularly finer sampling, and with very low background noise per image. Overall, the combination of ultrafine φ-slicing and smaller pixel size of EIGER enables more accurate measurement of reflection profiles in three dimensions.

#### 3.2.3. Effect of incomplete angular coverage in reciprocal space   

**Figure 9:**
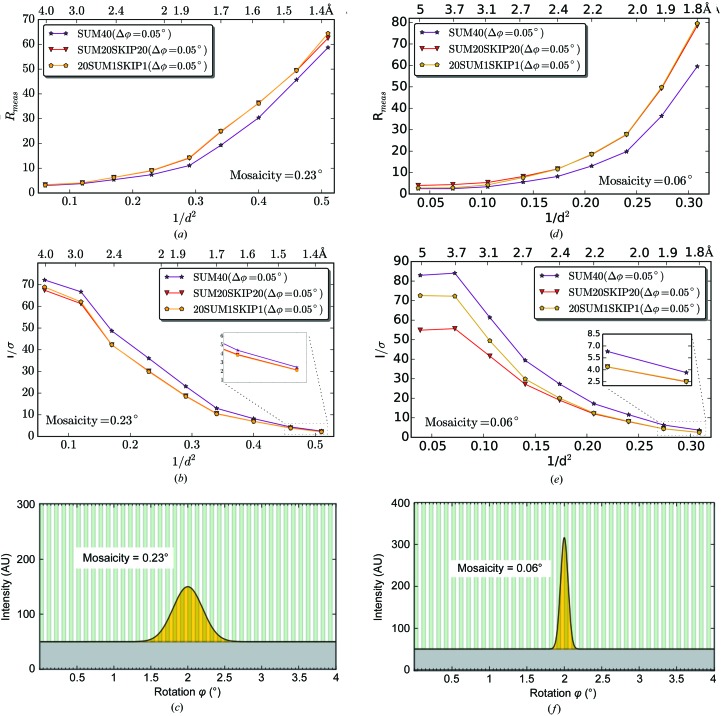
Effect of the systematic skipping of frames on data quality. Both lys_2 (*a*, *b*, *c*) and ins_1 (*d*, *e*, *f*) data sets were analyzed similarly. Taking SUM40 as a reference, the skipping of broad wedges of data (20 of every 40 frames; SUM20SKIP20) was compared with the skipping of thin wedges (one of every two frames and sum 20 together; 20SUM1SKIP1). (*c*) and (*f*) are a graphical illustration of the importance of good sampling of the rocking curve (mosaicity). The white bars represent the removed data in the case of SUM20SKIP20.

To gain a better understanding of the relationship between data quality and reflection profile (mosaicity), rotation angle and missing data during the dead time of the detector, the image summation was also combined with a systematic skipping of either broad or thin wedges of frames. Tests were carried out with both the lys_2 and ins_1 data sets, representing scenarios with large and small mosaicity, respectively. In both cases, summation of 40 frames of 0.00125° each, corresponding to 0.05° rotation per summed image, was taken as a reference. The lys_2_SUM20SKIP20 and lys_2_20SUM1SKIP1 data sets simulate 0.05° rotation data with half of the frames (data) removed in different manners. Comparisons of the skipping of broad wedges (lys_2_SUM20SKIP20) and thin wedges (lys_2_20SUM1SKIP1) including the control without skipping (lys_2_SUM40) are presented as *R*_meas_ and *I*/σ(*I*) values (Figs. 9 ▸ *a* and 9 ▸ *b* and Supplementary Table S5). As expected from counting statistics, skipping half the frames gives worse data-processing statistics across the whole resolution range. Interestingly, the two skipping schemes give very similar results. This is because with a mosaicity of 0.23° spot profiles are still well sampled along φ and the spot centroids and the spot profiles can be determined accurately even when broad wedges of images corresponding to 0.025° or 1/9 of the mosaicity have been removed. In Fig. 9 ▸(*c*), the broad wedge skipping is illustrated graphically. It is obvious that removing every 0.025° of data (represented by the white bars) still allows sufficient sampling of the reflection rocking curve. Therefore, further finer sampling with a 0.00625° interval as in 20SUM1SKIP1 only has a negligible effect.

The situation is different for the ins_1 data set, where the mosaicity is almost five times lower (0.05°). Here, the ins_1_SUM20SKIP20 data set is much worse than the ins_1_SUM1SKIP1 data set, particularly at low to medium resolution (Figs. 9 ▸ *d* and 9 ▸ *e* and Supplementary Table S5). Our explanation is that the reflection profile is significantly undersampled in the ins_1_SUM20SKIP20 data, where the missing wedge of 0.025° is about half of the mosaicity (Fig. 9 ▸ *f*). Although the net amount of removed data is the same for ins_1_20SUM1SKIP1, removing with 0.00625° intervals (1/10 of the mosaicity) ensures good sampling of the reflection profile and results in much improved data quality. This effect is more pronounced at low to medium resolution, where a significant part of the peak intensity could be missing in the broad wedge-skipping procedure.

#### 3.2.4. Optimal exposure and dose efficiency   

Radiation damage limits the amount of diffraction data which can be obtained from a given crystal volume. The radiation damage of protein crystals at cryogenic temperature has been well studied and tolerable dose limits range from 10 to 30 MGy (Henderson, 1990 ▸; Owen *et al.*, 2006 ▸). However, how much dose one should use for one data set is not easy to define. The ‘traditional burning’ strategy aims to obtain well recorded, high-resolution reflections on each diffraction image while keeping the accumulated dose within 10–30 MGy in one complete data set, which usually consists of 180° of rotation data. One alternative strategy is to distribute the total dose into multiple data sets (Liu *et al.*, 2011 ▸). The second strategy is particularly suitable when using readout noise-free X-ray detectors (Weinert *et al.*, 2015 ▸). With the capability of EIGER to keep a 99% duty cycle at high frame rate, skipping images systematically can effectively simulate data sets recorded with less exposure time, as long as the skipped angular range is much lower than the mosaicity and radiation damage is insubstantial. In the lys_2 (0.2 MGy) and ins_1 (0.25 MGy) examples, a half data set (*i.e.* by skipping every other frame) only results in a reduction of about 10% in *I*/σ(*I*) in the lowest resolution shell instead of a 30% reduction as expected from the counting statistics. This means that the observed *I*/σ(*I*) for the strong reflections is still largely limited by the instrumentation errors (Diederichs, 2010 ▸). Therefore, instead of one ‘full’ data set, two data sets with half the exposure time or half the beam transmission could improve the merged *I*/σ(*I*). From this perspective, such half-data simulation could be used to optimize the dose per data set to avoid unnecessary radiation damage for the next data set from the same crystal or from similar crystals.

In summary, data collection with EIGER is intrinsically fine φ-slicing. The exact slicing per frame depends on the rotation speed and the obtained slicing per image is determined by the auto-summation. A similar ultrafine φ-slicing data-collection method could be used with the PILATUS3 detector. However, only a 10 Hz frame rate could be used if one wants to keep the PILATUS3 duty cycle at 99%, which will make the experiment very long. When higher frame rates of 100 and 500 Hz are used, the corresponding duty cycles are 90 and 50% with PILATUS3, respectively. This misses 10 or 50% of the angular coverage in reciprocal space, the latter corresponding to the skipping of half the measured images discussed above. If the sampling with respect to the mosaicity of the crystal is too low, then this will significantly impair the data quality, as discussed for the insulin crystal in §[Sec sec3.2.3]3.2.3. Worse still, these ‘lost photons’ during detector readout still contribute to radiation damage.

### 3.3. Data collection at high rotation speed   

**Figure 10:**
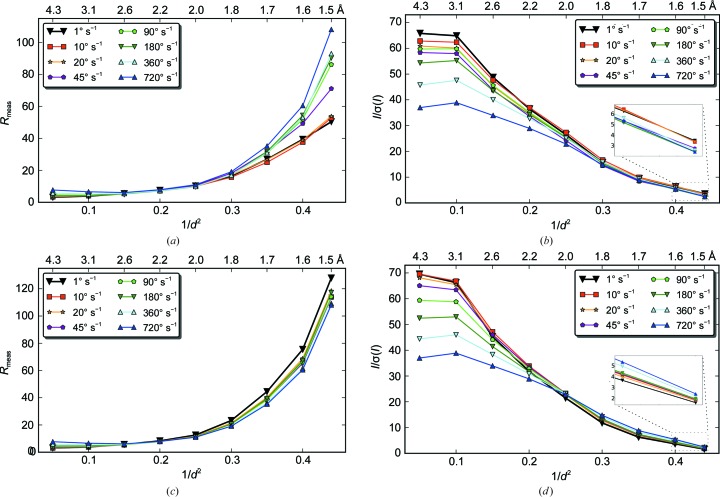
Data collection with higher rotation speeds. (*a*, *b*) A comparison of data sets collected from a lysozyme crystal at speeds between 1 and 720° s^−1^ is shown (lys_3 to lys_10). The detector was operated at a 800 Hz frame rate for all data sets and the beam transmission was adjusted to maintain the same overall dose per data set. (*c*, *d*) The same data sets used in Fig. 9 ▸ were reanalyzed by summing frames in each data set so that all data sets have an rotation angle of 0.9°. This allows the speed comparison to be made excluding the effects of the different rotation angles.

With microsecond dead time, EIGER should allow data collection with high rotation speed as long as the missing angular range during detector readout is significantly smaller than the crystal mosaicity. Data collection with rotation speeds of up to 720° s^−1^ were tested with an EIGER 1M operating at an 800 Hz frame rate with 20 µs dead time. The 20 µs corresponds to 0.00002, 0.0002, 0.0004, 0.0009, 0.0018, 0.0036, 0.0072 and 0.0144° missing data at angular speeds of 1, 10, 20, 45, 90, 180, 360 and 720° s^−1^, respectively. The lysozyme crystal used in this test has a mosaicity of 0.23°, which is about 16 times larger than the missing gap in the 720° s^−1^ experiment. Therefore, the missing wedge should not compromise the data quality much, as discussed in the previous section. The flux was adjusted such that each data set has the same accumulated X-ray dose (§2.3). The *R*_meas_ and *I*/σ(*I*) for data-collection speeds between 1 and 720° s^−1^ (data sets lys_3 to lys_10) are plotted in Figs. 10 ▸(*a*) and 10 ▸(*b*), and the numerical values are given in Table 2 ▸ and Supplementary Table S6. The data quality from the 1, 10 and 20° s^−1^ data sets is comparable, which implies that faster rotation up to 20° s^−1^ could be used in standard data collection.

Higher rotation speed results in progressively worse *R*_meas_ and *I*/σ(*I*) in both low- and high-resolution shells. Apparently, the high-speed experiment introduced additional measurement errors, which could come from goniometer imprecision at high rotation speed, high-frequency fluctuation of X-ray beam intensity and position, count-rate limitation of single photon-counting detectors and a relatively larger rotation angle per image. For weak reflections at high resolution, the count rate is well below the safe level of 50 Mcps mm^−2^ for a linear detector response and the *I*/σ(*I*) is determined by counting statistics and is relatively insensitive to instrumentation errors (Diederichs, 2010 ▸). Therefore, the deterioration in data quality at high resolution should be attributed to the increasing larger rotation angle used for data collection at higher speed. Indeed, at 20° s^−1^ the rotation angle of 0.025° is about 1/9 of the crystal mosaicity (0.23°), which is consistent with the recommendation from the image-summation study above and also explains the similarity in data quality when the rotation speed is below 20° s^−1^. As expected, when images with lower rotation speed were summed to simulate data with a 0.9° rotation angle, the high-resolution statistics of processed data for all rotation speed are very similar, as shown in Figs. 10 ▸(*c*) and 10 ▸(*d*).

For strong reflections at low to medium resolution, the significant drop in *I*/σ(*I*) and the asymptotic 〈*I*/σ(*I*)〉 ratio (ISa in Table 2 ▸) suggests various high-frequency instrumentation errors. In addition, the detector count-rate limitation could reduce *I*/σ(*I*) in high-speed data sets. In the high-speed series of data sets, the highest counts observed are less than 500 per pixel, which is below the 12-bit counter limit. In the 1° s^−1^ data set, medium to strong reflections have about 10–200 counts per spot. For ten counts per pixel, the count rate is 1.42 Mcps mm^−2^ [10 counts/0.00125 s/(0.075 mm)^2^]. In fast rotation experiments, a higher flux is needed to keep the total dose per data set the same, which means more diffracted photons in a shorter time: higher count rates. Therefore, the count rate for higher speed data sets could be approximately estimated by multiplying the ‘speed-up’ factor. The calculated count rates at different rotation speeds are plotted over the count-rate correction curve in Fig. 2 ▸. Up to 20° s^−1^, the count rate is within the linear response region. At 45 and 90° s^−1^, a correction has to be made for pixels with ten counts already and much more substantial correction is needed for pixels with more counts (*i.e.* stronger reflections). This can at least partially explain the start of degradation in data quality in the low-resolution shells. From 180° s^−1^ onwards, the count rate is beyond the correction limit for strong reflections and starts polluting the data quality towards medium resolution gradually. It is worthwhile noting that despite substantial count-rate limitation and other instrumentation errors, even the 360° s^−1^ data set, collected in just half a second, is still of decent quality as judged by a CC_1/2_ of 99.9% and can be used for structure refinement. The refined *R*_work_ and *R*_free_ are 0.195 and 0.249, respectively, for the 360° s^−1^ data and 0.182 and 0.233, respectively, for the 1° s^−1^ data. Note that the high count rates in fast rotation data could be handled better with PILATUS3 retriggering technology. However, the 1 ms dead time of the PILATUS3 detector results in a missing angle of 0.09° at a speed of 90° s^−1^, which will compromise data quality dramatically even for crystals with a relatively large mosaicity of 0.2°. Practically, this makes data collection at speeds greater than 90° s^−1^ impossible with any detector technology with a dead time longer than 1 ms.

### 3.4. 1 s native SAD phasing with EIGER 4M(16M)   

The success of native SAD phasing depends heavily on the precision and accuracy of diffraction data because the phasing signal comes from very small differences in diffraction intensities. To explore fast data collection for native SAD phasing, a 1 s native SAD experiment was attempted with an insulin crystal. A quadrant of the EIGER 16M detector was used to simulate an EIGER 4M(16M) detector. Diffraction data were collected with a 0.4° rotation increment at 160 Hz, which produced 64° of data in 1 s. Despite the large rotation angle compared with the crystal mosaicity of 0.23°, the additional background introduced by coarse slicing does not much compromise the data quality of the strong reflections at low to medium resolution, which contain substantial anomalous signal for experimental phasing. The substructure determination, density modification and phasing were straightforward with *SHELXC*/*D*/*E* *via* the *HKL*2*MAP* interface (Supplementary Fig. S1). The complete model was built automatically with *CRANK*2 and the final refined *R*_work_ and *R*_free_ are 0.208 and 0.256, respectively.

## 4. Conclusion and future prospects   

The introduction of the PILATUS detector in 2007 transformed data collection in macromolecular crystallography. The continuous and shutterless data-acquisition method and fine φ-slicing strategy were developed soon after. Since then, PILATUS detectors have been installed at many synchrotron MX beamlines worldwide. The EIGER technology has improved on the PILATUS3 generation detectors, with a smaller pixel size and a dead time between frames of as low as 3.8 µs, and offers a novel data-acquisition mode utilizing a combination of a kilohertz internal frame rate and frame summation, which extends the data depth significantly and keeps the duty cycle above 99%. Thus, the EIGER detector series represent the current state of the art of X-ray detectors for synchrotron applications. The fact that native SAD phasing was possible on a data set collected in 1 s demonstrates the high data quality that new EIGER detectors can produce at an unprecedented speed. EIGER’s high spatial resolution is especially useful in micro-crystallography and large-complex crystallography, where weak reflection spots need to be resolved and measured accurately.

Although fine φ-slicing with 1/2 of the mosaicity gives significantly better data than coarse slicing for PILATUS detectors, even finer slicing with 1/10 of the mosaicity provides even better results with the EIGER detector. We believe that the small pixel size of EIGER enables better sampling of reflections on the detector surface, which in turn helps to improve the angular sampling of reflections with the ultrafine φ-slicing method. This effect is likely to be prominent in micro-crystallography with low-mosaicity crystals using a micro-focused X-ray beam with low divergence, as promised at nearly diffraction-limited synchrotron sources, which are either under construction or at the planning stage worldwide. As an added benefit, an analysis of various summation and skipping schemes allows optimization of the rotation angle and exposure time using just one data set collected at a high frame rate, whose optimal settings could be applied to similar crystals.

The high frame rate and smaller pixel size of EIGER will lend itself to improvements in and beyond the standard rotation method of X-ray crystallography. High-speed data collection makes native SAD phasing faster by significantly reducing the total time needed to collect multi-orientation data sets (Weinert *et al.*, 2015 ▸; Olieric *et al.*, 2016 ▸). The high frame rate can further accelerate grid scanning to locate microcrystals or to locate the ‘sweet spot’ of large crystals (Aishima *et al.*, 2010 ▸; Bowler *et al.*, 2010 ▸; Zander *et al.*, 2015 ▸; Wojdyla *et al.*, 2016 ▸). One area that would particularly benefit from these advances is serial synchrotron crystallography (SSX), in which still images or a few degrees of rotation data are collected from hundreds or thousands of microcrystals and are merged together to obtain a complete data set (Gati *et al.*, 2014 ▸; Stellato *et al.*, 2014 ▸; Huang *et al.*, 2015 ▸, 2016 ▸). Two sample-delivery methods are widely used in SSX; one is injector-based and the other is goniometer-based. In the injector-based method, diffraction patterns are acquired while crystals pass through the X-ray beam (Botha *et al.*, 2015 ▸; Nogly *et al.*, 2015 ▸). Depending on the speed of the injector and the viscosity of the medium, crystals will exercise various motions, so data collection at kilohertz rates can record the motions in diffraction patterns and allow the best selection of useful images and correct treatment in data processing. In the goniometer-based method (also called the fixed-target and solid-support method), faster scanning could be used to either collect still diffraction images (Gati *et al.*, 2014 ▸; Coquelle *et al.*, 2015 ▸; Baxter *et al.*, 2016 ▸) or locate crystals (Zander *et al.*, 2015 ▸; Huang *et al.*, 2016 ▸) to subsequently pursue for dose-limited data collection from the best-diffracting crystals over a limited angular range. If SSX data sets are recorded at room temperature, where radiation damage is much more severe than at low temperatures, extremely fast data acquisition will allow better monitoring of the effects of radiation damage as it occurs (Huang *et al.*, 2015 ▸). Additionally, high-speed, high-flux data collection should allow more data to be collected on the time scale before damage manifests at room temperature (Owen *et al.*, 2014 ▸; Coquelle *et al.*, 2015 ▸). Exploring novel crystal-delivery methods such as acoustic levitation inherently depends on the availability of a fast detector like the EIGER 16M (Tsujino & Tomizaki, 2016 ▸).

Nearly 60 years after the very first X-ray crystal structure of a protein was determined, macromolecular crystallography is still evolving rapidly owing to the many improvements in X-ray sources, beamline instrumentation, automation, crystallization, sample delivery, data-collection and processing methods, and structure-determination software. We anticipate that the smaller pixel size, higher frame rate and negligible readout dead time of EIGER will not only enhance the quality and productivity of standard data collections but also foster the development of emerging data-collection techniques in experimental phasing, *in situ* and serial crystallography at X-ray synchrotron sources.

## Supplementary Material

PDB reference: lysozyme, collected at 1° rotation per second, 5lin

PDB reference: collected at 360° rotation per second, 5lio

PDB reference: insulin solved by native SAD from a data set collected in 1 s, 5lis

Supporting Information.. DOI: 10.1107/S2059798316012304/di5005sup1.pdf

## Figures and Tables

**Table 1:** Data-collection and processing statistics Values in parentheses are for the outer shell.

Data set	lys_1	lys_2†	ins_1†	ins_2‡
Wavelength (Å)	1.0332	1.0332	1.0332	1.5498
Temperature (K)	100	100	100	100
EIGER detector	1M	1M	1M	4M in 16M
Detector distance (mm)	50	50	60	130
Total rotation range (°)	180	180	90	160
Rotation range per image (°)	0.05	0.00125	0.00125	1
No. of images	3600	144000	72000	160
Exposure time (s)	0.05	0.00125	0.00125	0.00625
Flux (photon s^−1^)	7.2 × 10^9^	7.2 × 10^9^	1.8 × 10^10^	1 × 10^12^
Space group	*P*4_3_2_1_2	*P*4_3_2_1_2	*I*2_1_3	*I*2_1_3
Unit-cell parameters				
*a* (Å)	78.17	78.15	77.39	76.99
*b* (Å)	78.17	78.15	77.39	76.99
*c* (Å)	36.95	36.95	77.39	76.99
α (°)	90	90	90	90
β (°)	90	90	90	90
γ (°)	90	90	90	90
Mosaicity (°)	0.22	0.22	0.06	0.23
Resolution (Å)	1.4	1.4	1.81	2.3
No. of reflections	273655	273369	77145	17031
No. of unique reflections	22680	22683	7220	5453
Completeness (%)	98.2 (90.3)	98.2 (90.0)	99.7 (99.6)	83.1 (38.8)
Multiplicity	12.0 (5.8)	12.1 (5.8)	10.7 (10.2)	3.1 (1.5)
ISa	25.9	26.4	37.9	42.2
〈*I*/σ(*I*)〉	23.3 (2.6)	23.0 (2.5)	28.0 (3.7)	29.6 (3.0)
*R*_meas_ (%)	6.3 (55.4)	6.3 (58.7)	4.8 (58.6)	2.8 (24.4)
CC_1/2_ (%)	100.0 (75.5)	100.0 (75.0)	100.0 (91.3)	99.9 (91.9)

† The statistics for SUM40 are shown for the lys_2 and ins_1 data sets.

‡ The statistics are reported with Friedel pairs unmerged.

**Table 2:** Data-collection and processing statistics for the fast rotation experiment Values in parentheses are for the outer shell.

Data set	lys_3	lys_4	lys_5	lys_6	lys_7	lys_8	lys_9	lys_10
Wavelength (Å)	1.0332	1.0332	1.0332	1.0332	1.0332	1.0332	1.0332	1.0332
Temperature (K)	100	100	100	100	100	100	100	100
EIGER detector	1M	1M	1M	1M	1M	1M	1M	1M
Detector distance (mm)	50	50	50	50	50	50	50	50
Resolution (Å)	1.5	1.5	1.5	1.5	1.5	1.5	1.5	1.5
Total rotation range (°)	180	180	180	180	180	180	180	180
Rotation range per image (°)	0.00125	0.0125	0.025	0.05625	0.1125	0.225	0.45	0.9
No. of images	144000	14400	7200	3200	1600	800	400	200
Exposure time (s)	0.00125	0.00125	0.00125	0.00125	0.00125	0.00125	0.00125	0.00125
Beam transmission†	0.001	0.01	0.02	0.045	0.09	0.18	0.36	0.72
Rotation speed (° s^−1^)	1	10	20	45	90	180	360	720
Space group	*P*4_3_2_1_2	*P*4_3_2_1_2	*P*4_3_2_1_2	*P*4_3_2_1_2	*P*4_3_2_1_2	*P*4_3_2_1_2	*P*4_3_2_1_2	*P*4_3_2_1_2
Unit-cell parameters								
*a* = *b* (Å)	77.98	78.31	78.32	78.32	78.35	78.38	78.37	78.38
*c* (Å)	36.95	37.02	37.02	37.04	37.03	37.04	37.04	37.05
α = β = γ (°)	90	90	90	90	90	90	90	90
Mosaicity (°)	0.23	0.22	0.22	0.22	0.23	0.23	0.24	0.27
No. of reflections	248970	251327	251572	251909	251489	251726	253181	252979
No. of unique reflections	18845	19011	18994	18987	18965	18998	18979	18994
Completeness (%)	99.5 (97.9)	99.2 (96.1)	99.2 (96.1)	99.3 (96.4)	99.5 (97.6)	99.6 (98.5)	99.5 (98.1)	99.5 (98.2)
Multiplicity	13.2 (10.0)	13.2 (9.9)	13.2 (10.0)	13.3 (10.0)	13.3 (10.1)	13.3 (10.0)	13.3 (10.1)	13.3 (10.0)
ISa	24.2	22.4	22.1	21.2	21.9	19.3	15.4	11.9
〈*I*/σ(*I*)〉	23.8 (3.5)	23.8 (3.4)	22.7 (3.4)	21.5 (2.8)	22.2 (2.5)	21.3 (2.4)	20.1 (2.6)	17.6 (2.5)
*R*_meas_ (%)	6.9 (50.4)	6.8 (52.8)	7.2 (53.8)	7.5 (71.1)	7.4 (86.2)	7.7 (90.3)	8.6 (93.0)	10.2 (108.1)
CC_1/2_ (%)	100 (77.4)	99.9 (80.0)	99.9 (78.4)	99.9 (75.3)	99.9 (75.9)	99.9 (77.4)	99.9 (76.3)	99.8 (73.8)

† Beam transmission is presented as a fraction of the full beam (3.6 × 10^12^ photon s^−1^).
