# Supplementary material for: EIGER detector: application in macromolecular crystallography
Source: Acta Crystallogr D Struct Biol. 2016 Aug 31;72(Pt 9):1036–48. doi: 10.1107/S2059798316012304 (PMC5013597; doi:10.1107/S2059798316012304)
Supplement: Supplementary file 1 [file d-72-01036-sup1.pdf]

STRUCTURAL BIOLOGY

Volume 72 (2016)

Supporting information for article:

EIGER detector: application in macromolecular crystallography

Arnau Casanas, Rangana Warshamanage, Aaron D. Finke, Ezequiel Panepucci, Vincent Olieric, Anne Nöll, Robert Tampé, Stefan Brandstetter, Andreas Förster, Marcus Mueller, Clemens Schulze-Briese, Oliver Bunk and Meitian Wang

EIGER detector: application in macromolecular crystallography

Arnau Casanas[a], Rangana Warshamanage[a], Aaron D. Finke[a], Ezequiel Panepucci[a], Vincent Olieric[a], Anne Nöll[c], Robert Tampé[c], Stefan Brandstetter[b], Andreas Förster[b], Marcus Mueller[b], Clemens Schulze-Briese[b], Oliver Bunk[a], Meitian Wang[a]*

[a] Swiss Light Source, Paul Scherrer Institute, Villigen, CH-5232, Switzerland
[b] DECTRIS Ltd., Taefernweg 1, Baden-Dättwil, CH-5405, Switzerland
[c] Institute of Biochemistry, Biocenter, Goethe University Frankfurt, Max-von-Laue-Str. 9, D-60438 Frankfurt am Main, Germany

* Correspondence e-mail: meitian.wang@psi.ch

Table S1 Data processing statistics (XDS version March 1, 2015) for lys_1 and lys_1 2 × 2 binned data sets (Figure 4).

Resolution (Å)	R_{meas} (%)		$<I/\sigma(I)>$		$CC_{1/2}$ (%)	
	0.05° /0.05s	Binned 2x2	0.05° /0.05s	Binned 2x2	0.05° /0.05s	Binned 2x2
4.07	3.3	3.3	63.62	64.27	99.9	99.9
2.93	4.2	4.2	59.92	60.17	99.9	99.9
2.41	5.8	5.9	44.77	43.93	99.9	99.9
2.10	7.9	8.0	33.26	32.57	99.8	99.8
1.88	12.2	12.6	20.93	20.51	99.7	99.6
1.72	21.3	22.6	11.61	11.29	99.1	98.9
1.59	34.7	38.9	7.06	6.49	97.3	96.5
1.49	50.7	63.2	3.61	2.95	89.0	84.5
1.40	64.9	98.9	2.00	1.35	62.0	56.2
Total	6.7	7.2	20.96	20.46	99.9	99.9

Table S2 Data processing statistics (XDS version Oct 15, 2015) for lys_1 and lys_2 (SUM40) data sets (Figure 6).

Resolution (Å)	R_{meas} (%)		$<I/\sigma(I)>$		$CC_{1/2}$ (%)	
	0.05° /0.05s	SUM40	0.05° /0.05s	SUM40	0.05° /0.05s	SUM40
4.07	3.1	3.0	71.05	72.17	100	100
2.93	3.8	3.8	66.19	66.71	99.9	100
2.41	5.3	5.4	48.99	48.69	99.9	99.9
2.10	7.2	7.4	36.41	36.03	99.9	99.8
1.88	10.9	11.1	23.64	23.10	99.8	99.7
1.72	18.4	19.3	13.50	12.98	99.4	99.3
1.59	28.4	30.4	8.70	8.22	98.3	98.0
1.49	42.4	45.7	4.70	4.38	92.9	91.7
1.40	55.4	58.7	2.63	2.46	75.5	75.0
Total	6.3	6.3	23.28	23.04	100	100

Table S3 Data processing statistics (XDS version Oct 15, 2015) for different summation of frames in lys_2 data set (Figure 7).

Resolution (Å)	R_{meas} (%)					
	SUM5	SUM10	SUM20	SUM40	SUM80	SUM160
4.07	2.9	3.0	3.0	3.0	3.0	2.9
2.93	3.6	3.8	3.8	3.8	3.8	3.7
2.41	5.1	5.3	5.4	5.4	5.3	5.3
2.10	6.9	7.1	7.4	7.4	7.3	7.2
1.88	9.9	10.0	11.1	11.1	10.9	10.9
1.72	16.0	15.9	18.5	19.3	19.1	19.3
1.59	24.3	23.4	27	30.4	30.8	31.2
1.49	34.3	33.5	36.1	45.7	48.9	50.8
1.40	44.8	45.3	44.6	58.7	74.3	79.6
Total	5.8	5.9	6.2	6.3	6.3	6.4

Resolution (Å)	$<I/\sigma(I)>$					
	SUM5	SUM10	SUM20	SUM40	SUM80	SUM160
4.07	75.11	72.74	71.62	72.17	72.95	73.85
2.93	70.74	68.22	66.55	66.71	67.24	67.91
2.41	53.19	51.64	49.09	48.69	48.95	49.21
2.10	40.49	39.39	36.5	36.03	36.29	36.45
1.88	26.48	26.45	23.59	23.1	23.38	23.34
1.72	15.20	15.42	13.75	12.98	13.08	13.02
1.59	9.88	10.09	9.19	8.22	8.09	8.04
1.49	5.37	5.48	5.24	4.38	4.06	3.96
1.40	2.92	2.86	2.92	2.46	1.98	1.89
Total	25.5	24.99	23.57	23.04	23.08	23.15

Resolution (Å)	$CC_{1/2}$ (%)					
	SUM5	SUM10	SUM20	SUM40	SUM80	SUM160
4.07	100	100	100	100	100	100
2.93	99.9	99.9	99.9	100	99.9	99.9
2.41	99.9	99.9	99.9	99.9	99.9	99.9
2.10	99.9	99.9	99.8	99.8	99.9	99.9
1.88	99.8	99.8	99.7	99.7	99.7	99.7
1.72	99.4	99.4	99.2	99.3	99.2	99.3
1.59	98.1	98.2	97.9	98.0	98.0	98.0
1.49	92.5	93.6	92.9	91.7	91.9	92.0
1.40	77.3	80.3	77.9	75.0	75.9	73.7
Total	100	99.9	100	100	100	100

Table S4 Data processing statistics (XDS version Oct 15, 2015) for different summation of frames in ins_1 data set (Figure 8).

Resolution (Å)	R_{meas} (%)					
	SUM5	SUM10	SUM20	SUM40	SUM80	SUM160
5.10	2.5	2.5	2.5	2.6	2.6	2.5
3.73	2.4	2.4	2.5	2.5	2.5	2.5
3.08	3.2	3.3	3.3	3.3	3.4	3.5
2.68	5.3	5.4	5.5	5.4	5.5	5.7
2.41	7.8	7.9	8.1	8.1	8.3	8.9
2.20	11.9	12.4	12.6	12.7	13.3	14.4
2.04	17.6	18.1	19.1	19.5	20.5	22.8
1.91	29.2	31.7	34.0	35.9	38.6	44.3
1.81	42.4	47.2	53.5	58.6	64.3	73.9
Total	4.5	4.5	4.6	4.8	4.9	5.2

Resolution (Å)	$<I/\sigma(I)>$					
	SUM5	SUM10	SUM20	SUM40	SUM80	SUM160
5.10	86.37	83.61	81.63	82.12	82.01	82.75
3.73	87.98	84.95	82.95	83.25	83.00	83.57
3.08	65.50	62.79	61.22	61.01	60.43	59.82
2.68	42.70	41.72	39.65	39.16	38.40	37.44
2.41	30.23	28.79	28.04	27.59	26.84	25.68
2.20	19.59	18.51	17.98	17.59	16.83	15.80
2.04	13.68	12.67	12.16	11.92	11.41	10.54
1.91	7.83	7.22	6.71	6.44	6.08	5.46
1.81	4.98	4.53	4.05	3.73	3.46	3.06
Total	30.50	29.09	28.21	27.97	27.48	26.81

Resolution (Å)	$CC_{1/2}$ (%)					
	SUM5	SUM10	SUM20	SUM40	SUM80	SUM160
5.10	99.9	100	100	100	100	100
3.73	100	100	100	100	100	100
3.08	99.9	99.9	99.9	99.9	100	100
2.68	99.9	99.9	99.9	99.9	99.9	99.9
2.41	99.8	99.7	99.7	99.7	99.7	99.7
2.20	99.6	99.6	99.6	99.6	99.6	99.5
2.04	99.1	99.1	99.1	99.1	99.0	98.8
1.91	96.6	96.7	96.6	96.7	96.2	94.6
1.81	92.6	91.6	91.8	91.3	89.3	86.2
Total	99.9	100	100	100	100	100

Table S5 Data processing statistics (XDS version Oct 15, 2015) for different skipping frames of lys_2 and ins_1 data sets (Figure 9).

lys_2 data

Resolution (Å)	R_{meas} (%)			<$I/\sigma(I)$>			$CC_{1/2}$ (%)		
	SUM40	SUM20 SKIP20	20SUM 1SKIP1	SUM40	SUM20 SKIP20	20SUM 1SKIP1	SUM40	SUM20 SKIP20	20SUM 1SKIP1
4.07	3	3.3	3.2	72.17	67.44	68.9	100	100	100
2.93	3.8	4.1	4.1	66.71	61.26	62.08	100	99.9	99.9
2.41	5.4	6.3	6.3	48.69	42.23	42.3	99.9	99.9	99.9
2.10	7.4	9.0	9.1	36.03	30.10	29.89	99.8	99.8	99.8
1.88	11.1	14.1	14.3	23.10	18.63	18.48	99.7	99.6	99.5
1.72	19.3	24.8	25.0	12.98	10.51	10.39	99.3	98.7	98.6
1.59	30.4	36.3	36.2	8.22	6.98	6.93	98.0	96.2	96.4
1.49	45.7	49.4	49.5	4.38	3.90	3.83	91.7	86.5	87.2
1.40	58.7	62.5	64.4	2.46	2.15	2.11	75	64.8	60.8
Total	6.3	7.5	7.5	23.04	20.06	20.13	100	99.9	100

ins_1 data

Resolution (Å)	R_{meas} (%)			<$I/\sigma(I)$>			$CC_{1/2}$ (%)		
	SUM40	SUM20 SKIP20	20SUM 1SKIP1	SUM40	SUM20 SKIP20	20SUM 1SKIP1	SUM40	SUM20 SKIP20	20SUM 1SKIP1
5.09	2.5	4.0	2.8	82.96	54.86	72.57	100	99.9	100
3.72	2.5	4.4	2.9	84.05	55.64	72.26	100	99.9	100
3.07	3.4	5.4	4.3	61.33	41.56	49.41	100	99.9	99.9
2.68	5.6	8.2	7.7	39.41	27.19	29.85	99.9	99.8	99.8
2.40	8.2	11.7	11.6	27.30	19.00	20.04	99.8	99.5	99.5
2.20	13.1	18.4	18.6	17.24	12.00	12.38	99.6	99.1	98.9
2.04	19.8	27.7	27.9	11.59	8.02	8.20	99.2	98.1	98
1.91	36.4	49.3	49.8	6.31	4.36	4.38	96.7	92.8	93.7
1.80	59.5	78.5	79.6	3.65	2.49	2.54	91	84.6	82.9
Total	4.9	7.3	6.2	27.97	18.98	22.14	100	99.9	100

Table S6 Data processing statistics (XDS version Oct 15, 2015) for high rotation speed data sets lys_3 – 10 (Figure 10).

Resolution (Å)	R_{meas} (%)							
	1 (SUM5)	10	20	45	90	180	360	720
4.32	3.2	3.4	3.5	3.8	4.0	4.5	5.8	7.7
3.12	3.9	4.1	4.2	4.2	4.1	4.4	5.2	6.6
2.57	5.4	5.5	5.6	5.7	5.2	5.3	5.5	6.1
2.23	7.5	7.4	7.7	7.7	7.2	7.1	7.2	7.9
2.00	10.2	10.0	10.7	10.6	9.9	9.8	9.9	11.0
1.83	16.5	15.7	17.0	18.2	17.1	17.0	17.0	19.1
1.69	26.9	25.0	26.7	31.7	30.7	31.8	31.0	35.2
1.59	39.4	37.6	38.9	49.3	52.0	54.4	53.1	60.5
1.50	50.4	52.8	53.8	71.1	86.2	90.3	93.0	108.1
Total	6.9	6.8	7.2	7.5	7.4	7.7	8.6	10.2

Resolution (Å)	$\langle I/\sigma(I)\rangle$							
	1 (SUM5)	10	20	45	90	180	360	720
4.32	65.8	62.82	60.87	58.34	59.72	54.30	45.76	36.99
3.12	64.85	62.35	60.09	57.96	59.8	55.24	47.55	38.88
2.57	48.73	47.61	45.27	43.78	45.6	43.54	40.06	33.94
2.23	36.44	36.99	34.83	33.54	35.13	34.31	32.69	28.9
2.00	26.43	27.34	25.4	24.18	25.56	25.36	25.08	22.84
1.83	15.69	16.66	15.74	14.45	15.3	15.36	15.8	14.77
1.69	9.48	9.93	9.65	8.41	8.75	8.78	9.26	8.81
1.59	6.29	6.48	6.38	5.37	5.24	5.20	5.66	5.38
1.50	3.51	3.39	3.42	2.8	2.48	2.44	2.60	2.45
Total	23.80	23.81	22.72	21.45	22.20	21.30	20.06	17.56

Resolution (Å)	$CC_{1/2}$ (%)							
	1 (SUM5)	10	20	45	90	180	360	720
4.32	100	100	99.9	99.9	99.9	99.8	99.7	99.4
3.12	99.9	99.9	99.9	99.9	99.9	99.8	99.8	99.6
2.57	99.9	99.9	99.9	99.9	99.9	99.9	99.9	99.8
2.23	99.8	99.8	99.8	99.8	99.8	99.8	99.8	99.8
2.00	99.7	99.8	99.7	99.7	99.7	99.8	99.7	99.7
1.83	99.4	99.4	99.4	99.3	99.5	99.5	99.5	99.4
1.69	98.1	98.6	98.4	98.2	98.4	98.4	98.5	98.1
1.59	93.1	95.0	94.4	94.2	94.6	94.2	94.6	93.8
1.50	77.4	80.0	78.4	75.3	75.9	77.4	76.3	73.8
Total	100	99.9	99.9	99.9	99.9	99.9	99.9	99.8

lys_3 – 10 data sets processed with same 0.9° rotation angle

Resolution (Å)	R_{meas} (%)							
	1	10	20	45	90	180	360	720
4.32	3.1	3.1	3.2	3.5	4.0	4.6	5.9	7.7
3.12	3.8	3.8	3.8	3.9	4.2	4.6	5.4	6.6
2.57	5.7	5.4	5.5	5.5	5.4	5.6	5.7	6.1
2.23	8.3	7.8	7.9	7.8	7.8	7.8	7.8	7.9
2.00	12.6	11.4	11.8	11.3	11.3	11.2	11	11
1.83	23.3	20.9	21.4	20.7	20.7	20.7	19.5	19.1
1.69	44.5	38.6	40.2	38.5	38.3	39.4	36.1	35.2
1.59	75.5	66.7	68.8	65.3	66.2	67.3	61.7	60.5
1.50	127.9	114	118.5	113.5	114.1	117.2	109.2	108.1

Total	8.5	8.1	8.2	8.1	8.4	8.8	9.3	10.2

Resolution (Å)	$<I/\sigma(I)>$							
	1	10	20	45	90	180	360	720
4.32	69.54	69.57	68.09	65.05	59.39	52.44	44.41	36.99
3.12	66.24	66.81	65.44	63.43	58.79	52.95	45.99	38.88
2.57	45.25	47.19	46.25	45.97	44.12	41.44	38.33	33.94
2.23	31.82	33.86	33.15	33.5	32.84	31.64	30.82	28.9
2.00	21.45	23.27	22.65	23.18	23.13	22.75	23.14	22.84
1.83	11.81	13.07	12.67	13.13	13.28	13.35	14.22	14.77
1.69	6.28	7.09	6.87	7.15	7.35	7.38	8.16	8.81
1.59	3.69	4.18	4.02	4.25	4.32	4.37	4.99	5.38
1.50	1.64	1.89	1.81	1.92	1.97	2.00	2.26	2.45
Total	21.11	22.12	21.63	21.57	20.78	19.62	18.82	17.56

Resolution (Å)	$CC_{1/2}$ (%)							
	1	10	20	45	90	180	360	720
4.32	100	100	100	99.9	99.9	99.8	99.6	99.4
3.12	99.9	99.9	99.9	99.9	99.9	99.9	99.8	99.6
2.57	99.9	99.9	99.9	99.9	99.9	99.8	99.8	99.8
2.23	99.8	99.8	99.8	99.8	99.8	99.8	99.8	99.8
2.00	99.6	99.7	99.7	99.7	99.7	99.7	99.7	99.7
1.83	99.1	99.2	99.3	99.3	99.3	99.3	99.3	99.4
1.69	97.2	97.9	97.8	97.8	97.9	97.8	98.0	98.1
1.59	90.2	91.9	91.5	92.7	92.5	91.8	93.3	93.8
1.50	65.0	70.2	66.4	70.1	69.6	71.0	69.5	73.8
Total	99.9	99.9	99.9	99.9	99.9	99.9	99.9	99.9

Table S7 Data processing statistics (XDS version Oct 15, 2015) for one second native-SAD data set ins_2 (Suppl. Figure 1).

Resolution (Å)	R_{meas} (%)	$\langle I/\sigma(I)\rangle$	$CC_{1/2}$ (%)
6.81	2.2	62.86	99.9
4.85	2.1	59.52	99.9
3.97	2.2	55.75	99.9
3.45	2.6	47.17	99.9
3.08	3.9	32.67	99.8
2.82	5.9	19.53	99.6
2.61	9.4	10.91	99.8
2.44	13.4	6.05	97.3
2.30	24.4	2.99	91.9
total	2.8	29.59	99.9

Figure S1 One second native-SAD experiment. A quadrant of an EIGER 16M was used to simulate a EIGER 4M(16M) detector. A one second data collection time native-SAD experiment was attempted with an insulin crystal (data set insu_2). The CC_{all} versus CC_{weak} in SHELXD and contrast versus cycle of density modification in SHLXE are depicted.

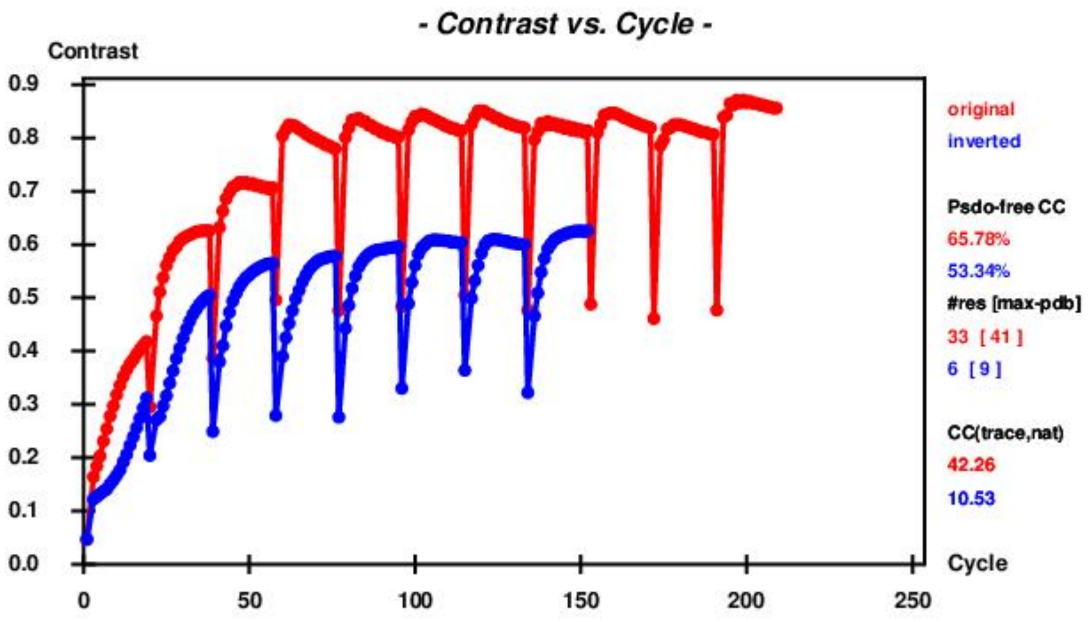

(a)

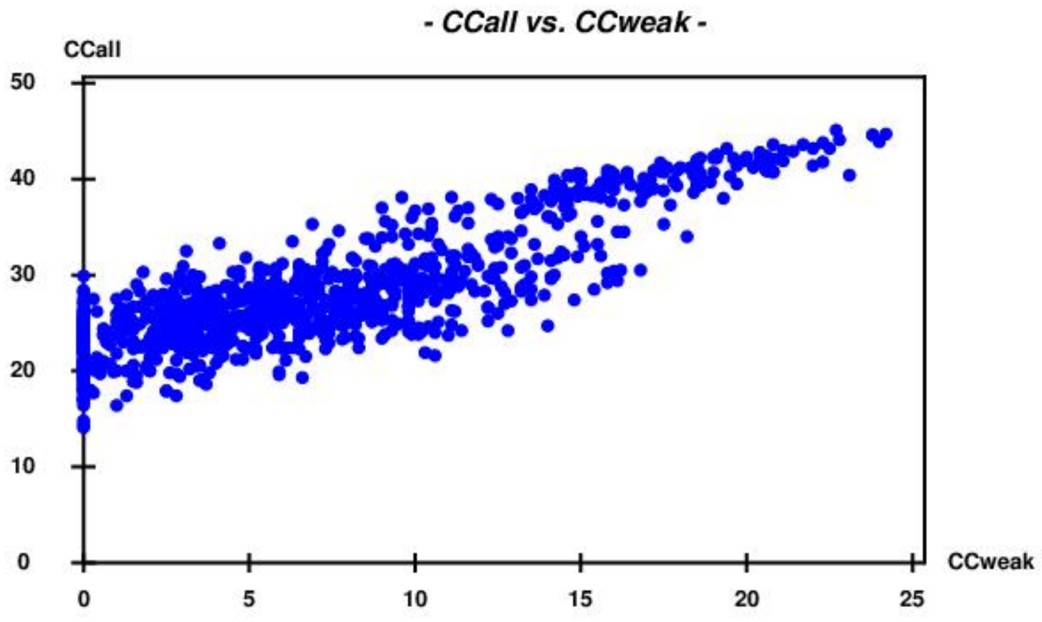

(b)
